# Remote Development of Symptomatic Intracranial Cavernous Malformation After Stereotactic Radiosurgery

**DOI:** 10.7759/cureus.21635

**Published:** 2022-01-26

**Authors:** Thomas T Patterson, Michael McGinity, Richard Crownover, Ramesh Grandhi

**Affiliations:** 1 Department of Neurosurgery, University of Texas Health Science Center at San Antonio, San Antonio, USA; 2 Department of Radiation Oncology, University of Texas Health Science Center at San Antonio, San Antonio, USA; 3 Department of Neurosurgery, University of Utah School of Medicine, Salt Lake City, USA

**Keywords:** cavernoma, radiation-induced, stereotactic radiosurgery

## Abstract

Cavernous hemangiomas, or cavernomas, are vascular malformations that affect about 0.1-0.5% of the population and usually result from sporadic or familial mutations of genes involved with endothelial cell junctions. They are histologically described as dilated vascular clusters, and they may occur in various areas of the body. Cavernomas of the central nervous system can generate localizing symptoms, including focal neurological defects, headaches, seizures, and hemorrhage. Radiation-induced cavernomas (RICs) have been described in the literature since 1994 and have been more frequently described in children. Although there has been speculation about the pathophysiology of RICs, no consensus exists in the literature, and pathological evaluation of RICs remains sparsely reported. We present the case of a 63-year-old patient who underwent stereotactic radiosurgery for treatment of an intracranial arteriovenous malformation (AVM) and subsequently developed a symptomatic RIC seven years later that required microresection. Clinicians should exercise diligence when monitoring patients with a history of intracranial radiation because of growing evidence supporting cavernomas as potential late-stage sequelae.

## Introduction

Cavernous hemangiomas (i.e., cavernomas, cavernous angiomas, or cavernous malformations) are vascular malformations characterized by dilated vascular channels and frequent infiltration into deep structures. Microscopic evaluation of cavernomas reveals a nonencapsulated structure with stroma infiltrated by large, thin-walled vascular spaces. Endothelial cell junctions are often abnormal to absent, suggesting a possible pathogenesis involving atypical cell-cell adhesion, cell polarity, and cell-extracellular matrix interaction [1]. Cavernomas affect 0.1-0.5% of the general population, with both sporadic mutations and familial forms having been identified. Inheritance is typically autosomal dominant, with a familial incidence rate of about 20% [1]. Loss-of-function mutations of ankyrin repeat-containing/cerebral cavernous malformation (KRIT1/CCM1), CCM2, and CCM3 gene loci on chromosome 7 have been indicated in the pathophysiology of cavernomas, possibly via a two-hit mechanism [2].

In patients with a cavernoma, symptoms may vary or be absent. Symptomatic clinical presentation of intracranial cavernomas correlates with the site of the lesion, often causing focal neurological deficits, partial or generalized seizures, cerebral hemorrhage, or headaches. Familial cavernomas may also affect the retina or skin, resulting in visual or cutaneous findings. Angiography is classically unable to identify cavernomas. Magnetic resonance imaging (MRI) is the most sensitive study for the detection of cavernomas within the neuraxis [2]. Management is typically driven by clinical status, with symptomatic patients usually undergoing resection and asymptomatic patients being monitored with observant management.

Reports of radiation-induced cavernomas (RICs) have been increasing [3-13]. Neoplasia after radiation exposure has been described for more than a century and is likely due to the proliferative effects of deoxyribonucleic acid (DNA) damage. Radiation has many molecular and biological effects linked to DNA changes, including nucleotide alterations, single- and double-strand breaks, and cross-linking. Ron et al. described radiation-induced neoplasms in a large retrospective cohort of nearly 11,000 subjects that had received broad scalp irradiation as children. The 30-year risk of developing a secondary neoplasm for these patients was 0.8%, with a mean interval of nearly 18 years after therapy until diagnosis. More recently, neoplasms induced by stereotactic radiosurgery (SRS) have been studied. In comparison to the low-dose, wide-field delivery of irradiation in traditional radiation therapy, SRS provides a high-dose pulse of radiation to a confined space of tissue. A review in 2013 estimated the risk of a patient developing a secondary neoplasm from SRS at 0.1-0.2% [14]; however, the correlation between SRS and cavernomas is less well quantified. The literature reports cavernomas after whole-brain radiotherapy (WBRT) and SRS, but analysis regarding radiation delivery method and dose is lacking [6, 10]. Herein, we present a case to add to a growing literature of RICs, with the intent to highlight the current understanding of RIC pathophysiology and histology, especially with regard to SRS.

## Case presentation

History and physical examination

**Figure 1:**
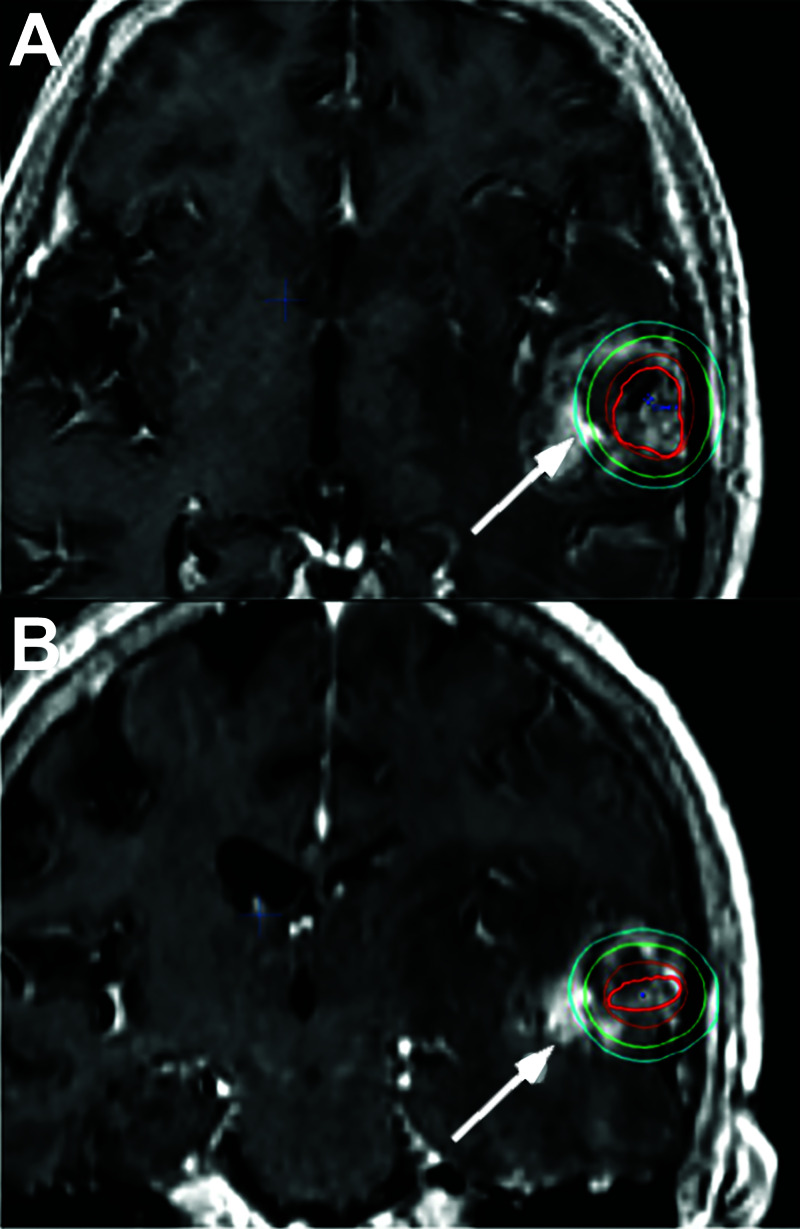
T1-weighted post-contrast magnetic resonance imaging axial (A) and coronal (B) scans from a patient before stereotactic radiosurgery (SRS) for arteriovenous malformation, superimposed with the radiotherapy doses (arrows) The left temporal lesion was targeted with a prescription dose of 20 Gy. The red ovoid line demonstrates the target of SRS. Isodense lines surrounding the target lesion represent a gradient of dose dissipation: 20 Gy (brown), 12 Gy (emerald), and 8 Gy (turquoise).

**Figure 2:**
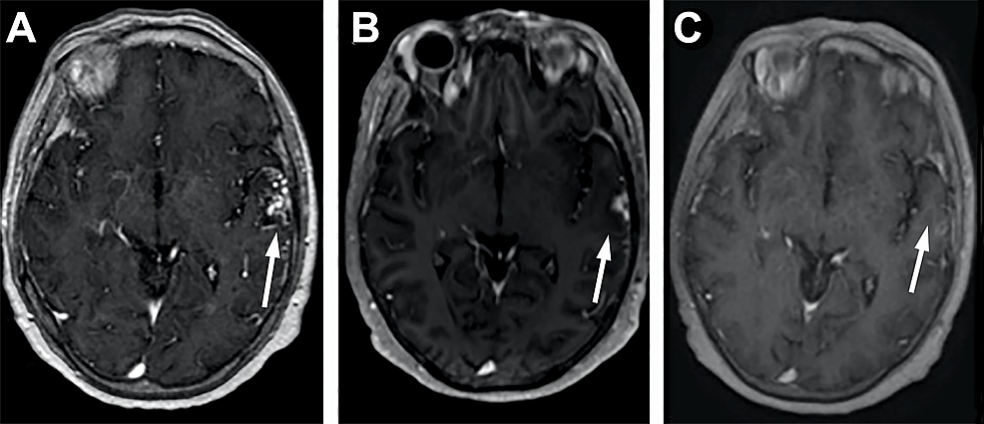
Serial T1-weighted post-contrast magnetic resonance imaging (MRI) scans indicating original presentation of left temporal arteriovenous malformation (AVM) and involution of lesion after stereotactic radiosurgery (SRS) (arrows) (A) MRI of the patient at the time of presentation for AVM. (B) MRI from 15 months post-SRS showing decreased size of AVM. (C) MRI from four years post-SRS with the resolution of AVM.

**Figure 3:**
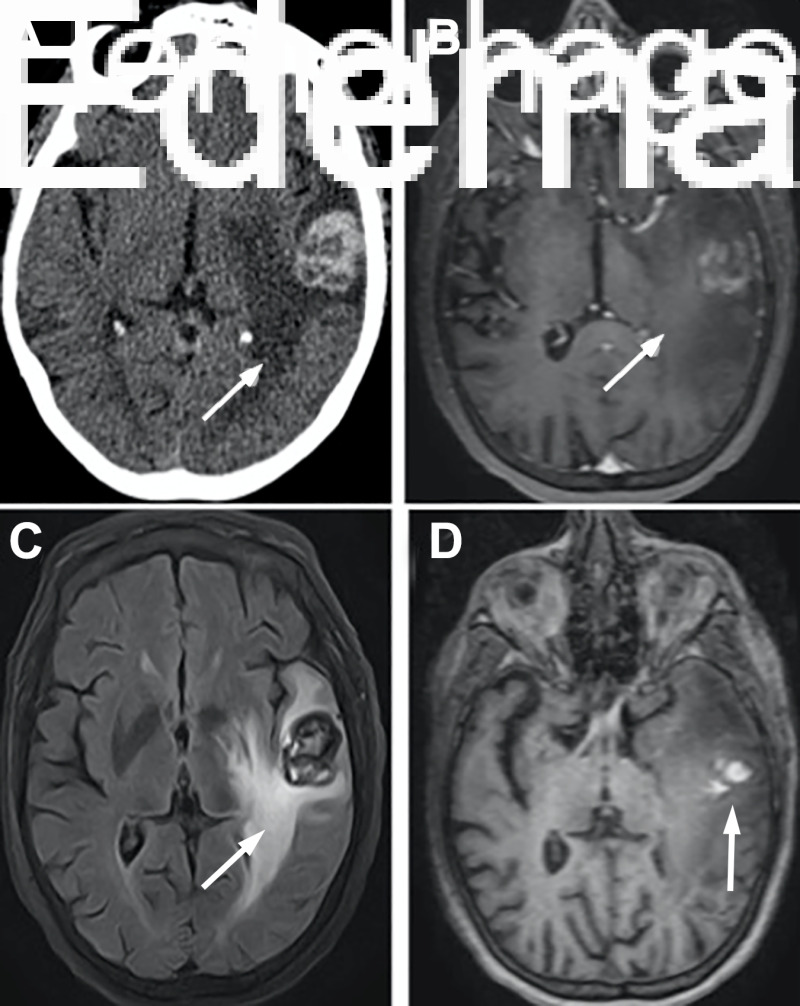
Head computed tomography scan and magnetic resonance imaging (MRI) scans (A) Non-contrast head computed tomography scan, (B) T1-weighted postcontrast enhanced magnetic resonance imaging (MRI), (C) T2 fluid-attenuated inversion recovery MRI demonstrating significant edema (arrows), and (D) T1-weighted precontrast MRI revealing 2.9-cm hyperdensity representing hemorrhagic mass (arrow) in the left temporal lobe found seven years after stereotactic radiosurgery.

A sixty-three-year-old, right-handed woman presented to the emergency department with chief complaints of progressive confusion and expressive aphasia over three days. Her past neurological history was significant for a Spetzler-Martin grade 2 left temporal arteriovenous malformation (AVM) that was treated seven years earlier with stereotactic radiosurgery using Novalis Tx (Varian Medical Systems Inc., Palo Alto, CA). The SRS therapy delivered a prescription dose of 20 Gy to the nidus of the lesion (Figure 1). Follow-up imaging demonstrated complete obliteration of the AVM (Figure 2); however, a non contrasted computed tomography scan of the head and MRI of the brain revealed a new left temporal hemorrhagic lesion (Figure 3).

Operation

A diagnostic cerebral angiogram revealed no discernible vascular malformation and confirmed the obliteration of the AVM. Given the suspicion that the cause of the intraparenchymal hemorrhage was a cavernoma, the patient was counseled regarding open surgical options. She decided to undergo an image-guided left temporal craniotomy under general anesthesia. Intraoperatively, a hemorrhagic mass was encountered with clear delineation between it and the surrounding brain tissue. The lesion was resected en bloc.

Pathological evaluation

**Figure 4:**
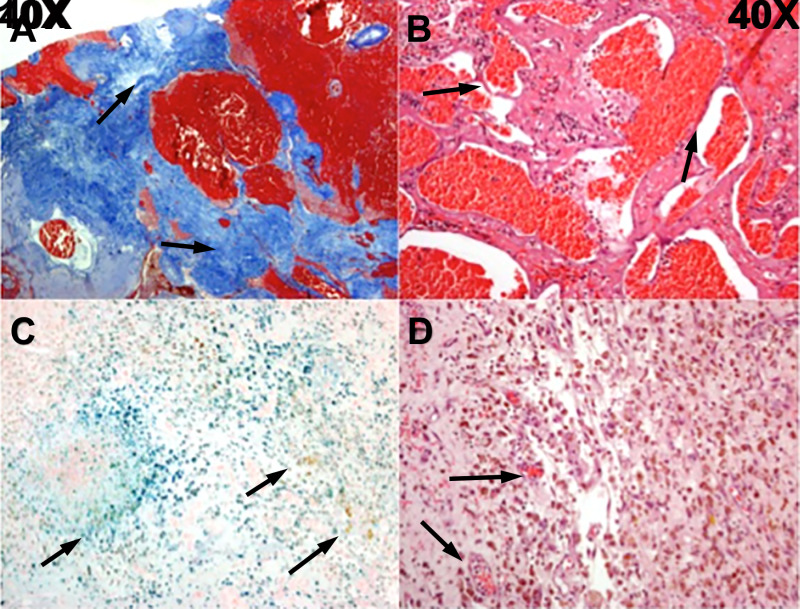
Histopathological evaluation of surgical specimen (A) Masson’s trichrome (10× magnification) demonstrating organized granulation tissue (arrows), probably secondary to prior radiation. (B) Hematoxylin and eosin (40× magnification) demonstrating thin-walled blood vessels (arrows) without intervening brain tissue. (C) Iron staining (40× magnification) demonstrating deposits of iron from multiple prior hemorrhages (arrows). (D) Hematoxylin and eosin (40× magnification) demonstrating siderophages (hemosiderin-containing macrophages) (arrows).

A pathological evaluation identified the hemorrhagic lesion as a cavernoma (Figure 4). A matrix of thin-walled blood vessels without elastin was present throughout the lesion without intervening brain tissue. Masson’s trichrome stain demonstrated areas of granulation tissue related to prior injury from radiation. Hemosiderin-containing macrophages (siderophages) were seen on hematoxylin and eosin staining. Iron staining demonstrated florid positivity.

Postoperative course

The patient was discharged to rehabilitation on postoperative day three with significant improvement in her speech. She remains neurologically intact with no speech difficulties or seizures.

## Discussion

Cavernomas are often thought to be the result of sporadic mutation or familial inheritance of the KRIT1/CCM1, CCM2, and CCM3 genes. In this case, we report a symptomatic cavernoma occurring seven years after stereotactic radiosurgery, which supports a growing collection of literature describing cavernomas as a distinct, late pathological consequence of intracranial radiation therapy. Ciricillo et al. [12] were among the first to describe cavernomas secondary to radiation while discussing “cryptic vascular malformations” in 1994. The pathophysiology is poorly understood, although parenchymal changes secondary to radiation have been described. Radiation injury to the brain broadly refers to a spectrum of findings, including but not limited to edema, demyelination, necrosis, and induced neoplasm [9]. Specifically, intracranial radiation injury is characterized by three periods of injury. Acute injury occurs in the immediate period surrounding the radiotherapy and is primarily attributed to edema caused by blood-brain barrier dysfunction [15]. The second period is referred to as early-delayed injury, which occurs from weeks to months after injury [15, 16]. This type of injury is thought to be caused by transient demyelination [15]. Late radiation injury effects occur months to years after radiation exposure. These late effects are typically the most profound and can be progressive, irreversible, and even fatal [16]. The changes surrounding the late phase include glial atrophy, vascular changes, and necrosis, which can be both focal and diffuse in nature [15, 16].

Radiation-induced neoplasms are a well-described phenomenon detailed as the delayed formation of meningiomas, central nervous system sarcomas, gliomas, and vascular proliferative lesions [7, 17, 18]. A set of criteria has been proposed to characterize radiation-induced neoplasms: 1) the lesion must occur in the field of previous radiation; 2) a prolonged (but undefined) period separates irradiation from lesion detection; and 3) the pathological characterizations of the primary and secondary lesions differ [18]. Formation of these neoplasms and vascular changes occur in the late phase after radiation. Although the mechanisms driving these changes are poorly understood, pathological examination of late radiation injury reveals arteriolar changes, including hyalinization and fibrinoid necrosis [16]. It has therefore been proposed that radiation-induced vascular changes may lead to vasculopathies, including the development of telangiectasias and cavernomas [9].

Several models have been put forth to describe the specific cause of cavernomas after radiotherapy. Radiation likely causes direct vascular damage and necrosis, with a delayed neovascular response. Tsao et al. [19] found that rats exposed to radiation experienced elevations in vascular endothelial growth factor (VEGF) expressivity, resulting in vascular changes manifested as a breakdown in the blood-spinal cord barrier. In 2002, Heckl et al. [11] published findings indicating that children exposed to radiation were at a greater risk than adults for developing cavernomas, noting that VEGF and hypoxia-inducible factor 1 (HIF-1) are more highly expressed in children. Thus, it is possible that the vasogenic factors VEGF and HIF-1 may be partially responsible for inducing vascular changes that result in cavernomas.

Another possible pathogenesis may be radiation-induced damage directly to DNA [9]. The identification of at least three genes involved in familial cavernomas supports the possibility that genetic damage from radiation therapy generates vascular changes resulting in a cavernoma. A study published in 2017 by Russo et al. [20] supported this theory by finding a novel KRIT1/CCM1 loss-of-function mutation in a patient presenting with microbleeds who was found to have RICs. The report highlights the role of the KRIT1 gene in cavernoma pathophysiology, suggesting radiation may induce and/or augment a predisposition for cavernomas. Carrier status for mutations in the CCM1, CCM2, or CCM3 genes might predispose patients receiving radiation therapy to developing cavernomas. In a study of the natural history of cavernomas, Moriarity et al. noted that patients with a positive family history of cavernomas more frequently had multiple lesions. Future avenues of study might examine whether RICs in patients with a positive family history also result in multiple lesions, which could further suggest a genetic component to the pathogenesis.

Interestingly, RICs in adults are less frequently described than RICs in children. In 2014, Ruggeri et al. [6] presented 86 cases of RICs. The mean age for presentation with RIC was 11.7 years old, and just seven patients (8.2%) were more than 40 years old. A 2015 article found only 23 adults among 146 identified cases of RIC, meaning pediatric cases accounted for 84% of those discussed [5]. Reports have noted that radiation-induced neoplasms demonstrate more profound effects in tissues with higher levels of proliferation, which are expectedly more widespread in pediatric tissue [17]. This might offer an explanation for the higher incidence of pediatric RICs. Alternatively, an increased VEGF expression in children compared with adults potentially results in a higher proportion of reported cases among younger individuals. Simpler mechanisms may instead underlie discrepancies in the age-related manifestation of RICs. Rates of survival length and adequate follow-up have the potential to mask the true incidence of RIC in the adult population. The time to lesion development is poorly defined as well. Ciricillo et al. [12] first reported a range of 1.5-16 years between radiation and detection of the lesion. Others suggest that the mean interval time is approximately 10-12 years, but wide variation in the interval time exists in the literature [3, 20]. Since the progression of RICs remains an unclear process, a slow-growing lesion may go undetected in the current monitoring of radiotherapy-treated adults who subsequently die of other causes. This may blur the incidence of this pathologic phenomenon.

Further factors remain to be explored. One such variable is the dose of radiation with regard to tissue damage and subsequent neoplasm. A positive, linear relationship has been demonstrated for Gy between 0.15 and 1.5 that correlates intracranial radiation with secondary lesions. It is well accepted that higher doses similarly induce pathological growth, although the extent is less clearly defined. In the case presented here, a prescribed dose of 20 Gy was delivered via SRS, which resulted in a secondary cavernoma. We do not currently know where this dose stands within the spectrum of those associated with other RICs, and thus it is still to be determined what dose is required to develop a RIC. In fact, our search found reports of inciting doses ranging from 1 to 60 Gy [3, 6, 14]. With so much variation in reported dosage, it is hard to pinpoint a threshold where radiation induces lesion development, so despite a suspected dose-dependent relationship, the precise correlation between dose and incidence in the context of RICs remains unknown.

Another component to consider is the use of SRS compared with WBRT. That is to say, the mechanism of delivery also likely contributes to lesion induction. Nagy et al. [3] recently reported that the estimated incidence of RIC after conventional radiotherapy is 3-4% at 10 years and 7-14% at 20 years, with a median detection time of 8-12 years after radiation. RIC after SRS is a less well-described phenomenon. A handful of reports have discussed RIC secondary to SRS, but the paucity of literature on this subject has previously hindered epidemiological study [8]. Only recently has the incidence of RIC after SRS been estimated. In a study of 425 patients receiving SRS, three patients developed subsequent RIC, producing an incidence of 0.9% at 15 years [3]. WBRT might increase the risk of RICs because of more parenchymal exposure to the radiation compared with SRS. The current literature does not describe a threshold dose or effectively compare SRS with WBRT. With few exceptions, the histopathology of RICs has rarely been discussed in the literature. Kleinschmidt-DeMasters and Lillehei [4] noted that two distinct pathological forms of RICs had previously been described: “cavernoma-like” and “coagulum-like” malformations. The cavernoma-like lesions appear histologically similar to spontaneous cavernomas. Coagulum-like lesions appear to result from organized hematomas and demonstrate interspersed thin-walled vessels and fibrinoid deposits [3]. These findings lead to a couple of notable conclusions. First, without previous uniform evaluation, the body of reported RICs may be misrepresentative of the true incidence. Additionally, because two subtypes have been described, multiple morphological changes are possible due to radiation. Whereas Kleinschmidt-DeMasters and Lillehei explain the “coagulum-like” subtype as a product of fibrinoid necrosis, the pathophysiology underlying the “cavernoma-like” lesions was not clear [4]. Analysis also failed to reveal any correlation between histological subtype and delivery method of radiation-WBRT versus SRS. These findings further highlight the uncertainty of both the incidence and mechanism regarding RICs.

## Conclusions

We present the case of a 63-year-old patient who underwent stereotactic radiosurgery for treatment of an intracranial arteriovenous malformation (AVM) and subsequently developed a symptomatic RIC seven years later that required microresection. RICs are increasingly acknowledged as a late consequence of radiation therapy. Because of the lack of consensus regarding interval time between radiotherapy and lesion presentation, a firm recommendation for standardized practice cannot be confidently established at this time. Regardless of the current understanding, clinicians should exercise diligence when monitoring patients with a history of intracranial radiation because of growing evidence that supports cavernomas-among other lesions-as potential late-stage sequelae with additional intervention required.

## References

[1] Haasdijk RA, Cheng C, Maat-Kievit AJ, Duckers HJ (2012). Cerebral cavernous malformations: from molecular pathogenesis to genetic counselling and clinical management. Eur J Hum Genet.

[2] Labauge P, Denier C, Bergametti F, Tournier-Lasserve E (2007). Genetics of cavernous angiomas. Lancet Neurol.

[3] Nagy G, McCutcheon BA, Giannini C, Link MJ, Pollock BE (2018). Radiation-induced cavernous malformations after single-fraction meningioma radiosurgery. Oper Neurosurg (Hagerstown).

[4] Kleinschmidt-DeMasters BK, Lillehei KO (2016). Radiation-induced cerebral vascular "malformations" at biopsy. J Neuropathol Exp Neurol.

[5] Narring M, Logak M, Brasme H, Gerber S, Zuber M (2015). Multiple radio-induced evolutive cavernomas in adult patients (Article in French). Prat Neruol - FMC.

[6] Ruggeri AG, Donnarumma P, Pichierri A, Delfini R (2014). Two cystic cavernous angiomas after radiotherapy for atypical meningioma in adult woman: case report and literature review. J Korean Neurosurg Soc.

[7] Chourmouzi D, Papadopoulou E, Kontopoulos A, Drevelegas A (2013). Radiation-induced intracranial meningioma and multiple cavernomas. BMJ Case Rep.

[8] Park YS, Kim SH, Chang JH, Chang JW, Park YG (2011). Radiosurgery for radiosurgery-induced cavernous malformation. World Neurosurg.

[9] Jain R, Robertson PL, Gandhi D, Gujar SK, Muraszko KM, Gebarski S (2005). Radiation-induced cavernomas of the brain. AJNR Am J Neuroradiol.

[10] Duhem R, Vinchon M, Leblond P, Soto-Ares G, Dhellemmes P (2005). Cavernous malformations after cerebral irradiation during childhood: report of nine cases. Childs Nerv Syst.

[11] Heckl S, Aschoff A, Kunze S (2002). Radiation-induced cavernous hemangiomas of the brain: a late effect predominantly in children. Cancer.

[12] Ciricillo SF, Cogen PH, Edwards MS (1994). Pediatric cryptic vascular malformations: presentation, diagnosis and treatment. Pediatr Neurosurg.

[13] Ron E, Modan B, Boice JD Jr, Alfandary E, Stovall M, Chetrit A, Katz L (1988). Tumors of the brain and nervous system after radiotherapy in childhood. N Engl J Med.

[14] Patel TR, Chiang VL (2014). Secondary neoplasms after stereotactic radiosurgery. World Neurosurg.

[15] Wong CS, Van der Kogel AJ (2004). Mechanisms of radiation injury to the central nervous system: implications for neuroprotection. Mol Interv.

[16] Valk PE, Dillon WP (1991). Radiation injury of the brain. AJNR Am J Neuroradiol.

[17] Kumar PP, Good RR, Skultety FM, Leibrock LG, Severson GS (1987). Radiation-induced neoplasms of the brain. Cancer.

[18] Marus G, Levin CV, Rutherfoord GS (1986). Malignant glioma following radiotherapy for unrelated primary tumors. Cancer.

[19] Tsao MN, Li YQ, Lu G, Xu Y, Wong CS (1999). Upregulation of vascular endothelial growth factor is associated with radiation-induced blood-spinal cord barrier breakdown. J Neuropathol Exp Neurol.

[20] Russo A, Neu MA, Theruvath J (2017). Novel loss of function mutation in KRIT1/CCM1 is associated with distinctly progressive cerebral and spinal cavernous malformations after radiochemotherapy for intracranial malignant germ cell tumor. Childs Nerv Syst.

